# Supplementary material for: Gaps, challenges and opportunities towards achieving the 95-95-95 targets in Cameroon: A systematic review and meta-analysis protocol
Source: PLoS One. 2025 Feb 10;20(2):e0317850. doi: 10.1371/journal.pone.0317850 (PMC11809780; doi:10.1371/journal.pone.0317850)
Supplement: S2 File — (DOCX) [file pone.0317850.s002.docx]

**Additional file 2**: Search Strategy

| Database | Query |
| --- | --- |
| PubMed | (((HIV Infections[MeSH] OR HIV[MeSH] OR hiv[tw] OR hiv-1*[tw] OR hiv-2*[tw] OR hiv1[tw] OR hiv2[tw] OR hiv infect*[tw] OR human immunodeficiency virus[tw] OR human immunedeficiency virus[tw] OR human immuno-deficiency virus[tw] OR human immune-deficiency virus[tw] OR ((human immun*) AND (deficiency virus[tw])) OR acquired immunodeficiency syndrome[tw] OR acquired immunedeficiency syndrome[tw] OR acquired immuno-deficiency syndrome[tw] OR acquired immune-deficiency syndrome[tw] OR ((acquired immun*) OR (deficiency syndrome[tw])) OR (sexually transmitted diseases, viral"[MeSH]) AND (diagnosis"[MeSH] OR diagnostic[tw]) AND ((therapy)[Subheading] OR (therapeutics)[MeSH] OR (Treatment[tw])) OR (antiviral agents) [All Fields] OR "antiviral agents"[MeSH] OR Antiviral Agents[tw] AND ((viral load) [MeSH Terms] OR (viral-load) OR (viral suppression) OR (viral control) OR (viral response) [tw] OR su ppression[All Fields] AND ("virology"[MeSH Terms] OR viral[tw]) AND ("Cameroon"[MeSH] OR Cameroon[tw] OR Cameroon [Title/Abstract] ) |
| Embase  OvidSP | 1. exp antiretrovirus agent/  2. exp highly active antiretroviral therapy/  3. ((viral control) OR (viral responce)) tw  4. or/1-3  5. exp Human immunodeficiency virus infection/  6. exp ((health disparate) OR (minority and vulnerable populations) OR (minority population))tw  7. (HIV OR HIV 1 OR HIV 2 OR human immunodeficiency virus OR human immuno-deficiency virus OR (human immuno deficiency virus) OR acquired immunodeficiency syndrome OR acquired immuno-deficiency syndrome OR (acquired immuno deficiency syndrome)).tw.  8. or/6-7  9. "HIV diagnosis " OR "AIDS diagnosis" tw.  10. "therapy" OR therapeutics OR “Treatment” OR "antiviral agents” OR antiretroviral “.tw  11. 5 or 8  12. 9 - 11  13. exp Cameroon/  14. Adamawa OR Center OR Est OR Far North OR Littoral OR North OR Northwest OR West OR South OR South west. tw  16. 12 or 13 or 14  17. 12 and 16  18. limit 17 to yr="2017 - 2023" |
| CINAHL | S1 (MH "diagnosis")  S2 (MH " health disparate ")  S3 (MH "Antiviral Agents")  S4 TI ( (anti and HIV) OR antiretroviral* OR (anti and retroviral*) OR HAART OR cART OR (anti and acquired immunodeficiency) OR (anti and acquired immunedeficiency) OR (anti and acquired immuno-deficiency) OR (anti and acquired immun* and deficiency) ) OR AB ( (anti and hiv) OR antiretroviral* OR (anti and retroviral*) OR HAART OR cART OR (anti and acquired immunodeficiency) OR (anti and acquired immunedeficiency) OR (anti and acquired immuno-deficiency) OR (anti and acquired immun* and deficiency)  S5 S1 OR S2 OR S3 OR S4  S6 (MH "HIV diagnosis ")  S7 (MH " antiretroviral ")  S8 TI ( HIV OR HIV 1 OR HIV 2 OR human immunodeficiency virus OR human immuno-deficiency virus OR acquired immunodeficiency syndrome OR acquired immunodeficiency syndrome OR (acquired immuno-deficiency syndrome) ) OR AB ( HIV OR HIV 1 OR HIV 2 OR human immunodeficiency virus OR human immuno-deficiency virus OR acquired immunodeficiency syndrome OR acquired immuno-deficiency syndrome or (acquired immuno deficiency syndrome)  S9 (MH "Sexually Transmitted Diseases, Viral+")  S10 S6 OR S7 OR S8 OR S9  S11 TI ("viral load " OR "viral suppression AND "virology suppression" OR viral-load )  S13 S5 OR S10  S14 S11 AND S13  S15 (MH "Cameroon+")  S16 TI (Adamawa OR Center OR Est OR Far North OR Littoral OR North OR Northwest OR West OR South OR South west).  S17 S14 OR S15 OR S16 |
