# Supplementary material for: Gaps, challenges and opportunities towards achieving the 95-95-95 targets in Cameroon: A systematic review and meta-analysis protocol
Source: PLoS One. 2025 Feb 10;20(2):e0317850. doi: 10.1371/journal.pone.0317850 (PMC11809780; doi:10.1371/journal.pone.0317850)
Supplement: Attachment — Submitted filename: Responses to reviewers.docx [file pone.0317850.s005.docx]

**Responses to Editor’s Comments:**

Academic Editor Comments:

1. Describe how you plan to handle translation bias from the French articles, in addition to the English.

**Response 1**: We thank the Editor for this comment. However, there will not be any translation bias from English to French articles/reports as both languages are adopted at national level and are perfectly mastered by all co-authors. In effect some authors have English as first-language for expression whereas others have French as first-language for expression. We have added a comment on that light (see lines 130-132, page 5).

2. Overall, this is not a systematic review to address SWOT towards 95-95-95, rather this is a protocol to conduct the review hence the objective of this protocol is to provide a standardized document to the scientific community for future use for similar review.

**Response 2**: We thank the Editor for this comment. We have revised and clarify the aim of this protocol accordingly (see line 39, page 2; and lines 88-89, page 3).

3. Include the potential limitation of this protocol, indicated below, or clarify in the manuscript how you plan to mitigate the issues:

- Data Availability: There may be limitations in the availability or quality of data reported in the included studies, which could affect the ability to perform robust meta-analysis.
- Heterogeneity: High variability between studies in terms of design, methodology, and participant characteristics could impact the synthesis of results and the ability to draw generalizable conclusions.
- Publication Bias: The review may be influenced by the publication bias of including only published studies and conference abstracts, which might not represent all relevant data.
- Quality Assessment: The assessment of the quality and risk of bias in studies could be subjective and dependent on the tools used (e.g., ROBINS-I, ROBIS). Variability in assessment could affect the interpretation of the evidence.
- Context-Specific Findings: The findings of the review will be context-specific to Cameroon, which may not be generalizable to other regions or countries with different healthcare systems and epidemiological profiles.
- Protocol Rigidity: The protocol might be rigid and not easily adaptable to new evidence or changes in the research landscape. Updates to the protocol or search strategy might be necessary to address emerging data or shifts in the field.
- Resource Limitations: The review might face constraints related to time, resources, or access to certain databases and literature, which could affect the comprehensiveness of the review.

**Response 3**: We thank the Editor for this comment. We have revised our protocol limitation exactly as recommended (see lines 247-271, page 8).

*Once again, we thank the Editor for the constructive remarks and are very much confident that the current version is suitable for publication. All other comments in the manuscript have also been addressed accordingly (see lines 104-105, page 4; and lines 129-130, page 5).*
